# Social Avoidance and Social Adjustment in Chinese Preschool Migrant Children: The Moderating Role of Household Chaos and Gender

**DOI:** 10.3390/ijerph192416769

**Published:** 2022-12-14

**Authors:** Jingjing Zhu, Xiaoqi Yin, Guangheng Wang, Yaoqin Jiang, Yan Li

**Affiliations:** 1 Shanghai Institute of Early Childhood Education, Shanghai Normal University, Shanghai 200234, China; 2 Changning Institute of Education, Shanghai 200050, China; 3 Punan Kindergarten, Shanghai 200126, China

**Keywords:** social avoidance, household chaos, social adjustment, migrant preschoolers, China

## Abstract

The present study explored the moderating role of household chaos and gender in the relation between social avoidance and social adjustment among Chinese preschool migrant children. Participants were 148 children (82 boys, M_age_ = 62.63 months, SD = 0.05) from two kindergartens, Shanghai, People’s Republic of China. Multi-source assessments included: (1) mother ratings of children’s social avoidance; (2) mother ratings of families’ household chaos; (3) teacher ratings of children’s prosocial behavior, peer exclusion, interpersonal skills, and internalizing problems. Results showed that social avoidance significantly predicted peer exclusion among Chinese migrant preschoolers. Moreover, household chaos moderated the relationship between social avoidance and social adjustment. Specifically, at higher levels of household chaos, social avoidance was negatively associated with interpersonal skills. In contrast, social avoidance was not associated with interpersonal skills at a lower level of household chaos. In addition, social avoidance was positively associated with peer exclusion among boys but not girls. The current findings inform us of the importance of reducing household chaos to buffer the negative adjustment among socially avoidant young children who migrated from rural to urban China. The findings also highlight the need to pay particular attention to migrant socially avoidant boys’ development in early childhood and the importance of considering the meaning and implication of social avoidance for migrant preschoolers in Chinese culture.

## 1. Introduction

Since the reform and opening of the People’s Republic of China in the 1980s, under the guidance of relevant national policies and with the acceleration of urbanization, the rural population has continued to move to cities and developed areas, forming a large group of migrant population. More than 236 million people migrated from rural to urban areas [1]. In the migration process, children who move with their parents become migrant children. Migrant children can be divided into preschool migrant children, compulsory education migrant children, and older children [2]. From 2000 to 2015, the number of preschool migrant children aged 3 to 5 increased from 3.77 million to 5.9 million, accounting for 17.2% of the total number of migrant children in that year, an increase of 56.5% [3]. Thus, we cannot ignore preschool migrant children.

Based on Embedding and Disembodying Theory [4], migrant children are at risk of being under-embedded in the urban environment. They constantly face new and unfamiliar territory [5]. Previous studies have found that migrant children are more likely to exhibit social withdrawal behaviors [6], internalizing problems, and externalizing problems [7]. Therefore, it is necessary to identify the social adjustment of preschool migrant children, and the positive factors that may be conducive to promoting the social adjustment of preschool migrant children.

Social withdrawal is the behavior in which children habitually inhibit their participation in group activities and peer interactions, spending more time to be left alone [8]. According to Asendorpf’s theoretical model of social approach–avoidance motivations, children’s social withdrawal behavior is determined by a combination of social approach motivations (i.e., desire to seek social interactions) and social avoidance motivations (i.e., desire to avoid social interactions), which can be further classified as shyness, unsociability, and social avoidance [8,9].

Shyness is the combination of high approach motivation and high avoidance motivation, referring to behaviors in which children want to interact with others but are afraid to come forward to participate because of wariness and anxiety [10]. From early childhood to adolescents, shyness was associated with a range of adverse outcomes (e.g., internalizing social anxieties, and peer exclusion) in the West [11,12]. Unsociability is the combination of low approach motivation and low avoidance motivation, referring to behaviors in which children prefer to be alone and are not anxious to interact with peers [13]. Compared with shyness, unsociability is seen as a relatively benign form of social withdrawal [8,14]. Research has shown that unsociable adolescents and children do not manifest defects in social skills internalizing problems or peer difficulties [11,13]. Social avoidance is the combination of low approach and high avoidance motivation, referring to behaviors in which children seek to stay alone and actively avoid social interaction [9]. Researchers speculated that socially avoidant children might have the most significant risk of adjustment difficulties [9,15]. However, social avoidance remains relatively understudied [16,17]. In a sample of Western early adolescents, social avoidance was associated with a wide range of negative outcomes, including peer difficulties, internalizing problems (e.g., depression, overanxious, and separation anxiety), and externalizing problems (e.g., overt and relational aggression, conduct trials, and oppositional defiance) [18,19]. Coplan et al. [20] found that social avoidance was associated with peer exclusion after controlling for shyness and unsociability in a sample of Canadian young children.

### 1.1. Social Avoidance in China

Cultural contexts influence the socio-emotional functioning of the different substrates of social withdrawal. The same behavior may lead to a different social adjustment in different cultural contexts [21]. In the traditional culture of China, children who are cautious, behaviorally inhibited, and self-restrained are encouraged and viewed as “well-behaved” children of social maturity, mastery, and understanding [21,22]. Thus, social withdrawal would not be viewed as maladaptive in Chinese culture. For example, Chen et al. [23] found that shyness was positively associated with sociability-leadership and peer acceptance in Chinese children.

However, influenced by Western culture that encouraged self-expression, and with the extensive economic and social reforms in China, people tended to change their views on withdrawal behavior, especially in urban areas [16]. Indeed, recent studies in China found that shyness has changed from a positive trait to a negative trait in urban cities, which was revealed to be associated with adjustment difficulties (e.g., peer difficulties and internalizing problems) [24,25]. Furthermore, unsociability was related to many adjustment problems, such as peer difficulties, learning difficulties, and psychological disorders (i.e., depression and loneliness) [25,26]. Moreover, social avoidance is the subtype at the most risk of social adjustment [9,15]. Indeed, some evidence showed that social avoidance posed a threat of maladjustment among Chinese youth in the limited empirical consideration [27]. For example, in early adolescents, social avoidance positively predicted peer relationship problems, loneliness, and lower peer acceptance in later development [27,28]. Compared with other subtypes of social withdrawal, social avoidance is associated with internalizing problems (e.g., depression, loneliness, social anxiety) and peer difficulties in adolescents [16,29]. 

Indeed, the Chinese cultural system emphasizes collectivism, interdependence, and social affiliation [28], and the behaviors of spending more time alone (i.e., separating oneself from the group) go against Chinese cultural norms of group affiliation [21]. Thus, all forms of withdrawn behavior may associate with negative outcomes, as it is regarded as selfish to avoid social interaction by removing oneself from the peer group [30]. As aforementioned, shyness and unsociability were associated with maladjustment (e.g., internalizing and externalizing problems, and peer difficulties) among Chinese children and adolescents [20,24,25]. Based on previous studies, it is known that social avoidance may have worse social adaptation than the other two subtypes of social withdrawal (i.e., shyness and unsociability) in the Chinese cultural context [17]. Although most of the existing literature about social avoidance focused on adolescents and older children, several pieces of evidence showed that socially avoidant preschool children would meet the most negative attitudes from peers [31], and social avoidance was associated with asocial behavior, peer difficulties, and anxiety [17,32].

In conclusion, most of the existing literature generally points to school-age children. Not much attention has been paid to social avoidance and social adjustment characteristics of preschool children aged 3–6 years, not to mention preschool migrant children in China. Therefore, it is necessary to explore social avoidance in Chinese migrant preschoolers to expand previous studies and gain a broader understanding of the influences of social avoidance. 

### 1.2. Moderating Effect of Household Chaos

Household chaos is a family environment characterized by noise, crowding, and a lack of order and routine [33]. According to the Ecological System Theory, family is one of the primary microsystems that have the most significant impact on children’s development, and household chaos is one of the most important physical characteristics in family environments [34]. Thus, as a direct source of influence, higher levels of household chaos were associated with a more negative social adjustment of children [35]. For instance, in samples of children and adolescents, household chaos was associated with lower IQ, conduct problems, and negative socioemotional adjustment [36,37], and the noise in the family would make adolescents feel crowded, tense, annoyed, and uncomfortable, and have more competitive behaviors [38]. Higher levels of household chaos (e.g., instability, lack of routine, and family noise) were related to more internalizing and externalizing problems and lower receptive vocabulary scores, even in early childhood [39,40]. In a sample of young Chinese children, household chaos was also associated with internalizing and externalizing problems [41].

Moreover, compared to urban non-migrant children in China, migrant children generally live in the suburbs or old towns of urban areas, where the nature of housing is temporary, and the quality of housing is generally poor [42,43]. Thus, preschool migrant children may face greater household chaos in China [43]. According to Emotional Security Theory, children’s adjustment and development are influenced by their feelings of security in the family system [44]. Researchers argued that high household chaos impaired parent–child interpersonal relationships [45], and was associated with more parental negativity, less parental warmth, and more stressful events [36]. The emotional distress and social anxiety of socially avoidant children may be heightened when emotional security within the family is negative due to exposure to high household chaos.

In addition, according to the Diathesis-Stress Model, children with certain vulnerability characteristics (e.g., irritable, uptight) would be more affected by adverse environmental factors and exhibit more developmental difficulties [46,47,48]. For example, family adversity (e.g., financial stress, marital conflict, and parenting overload) was not related to prosocial behavior in low-reactive kindergarteners. However, the association was negative for high-reactive kindergarteners [49]. Household chaos is a “negative environment factor” for children. Coe et al. [50] reported that higher levels of household chaos were associated with more punitive discipline practices, which then lead to a greater number of severe externalizing problems of children. Similarly, Wang et al. [41] found that high levels of household chaos increased the risk of externalizing problems in children who had experienced parental rejection. In this regard, social avoidance is a characteristic of “vulnerability”, and socially avoidant children tend to respond to stressful social situations with greater reactivity and emotional volatility [51]. Thus, socially avoidant children may be more sensitive to higher levels of household chaos and would show more negative social adjustment.

To summarize, it seems reasonable to argue that migrant socially avoidant preschoolers may face greater household chaos in China. Based on the previous studies, the present study hypothesized that household chaos would moderate the relationship between social avoidance and social adjustment in preschool migrant children in China. Specifically, higher levels of household chaos may be a risk factor exacerbating social maladjustment in Chinese migrant socially avoidant preschoolers.

### 1.3. Moderating Effect of Gender

Gender plays a significant role of children’s social adjustment. Specifically, compared to girls, boys were less empathetic and friendly and had worse social skills [52]. For example, a study of 101 Chinese preschoolers (48 boys) found that boys had significantly more externalizing behavior problems than girls [53].

Moreover, evidence found that gender differences related to the implications of social withdrawal; specifically, as compared to girls, socially withdrawn behaviors may cause more negative implications for boys because such behaviors violate the gender norm of male social assertion and dominance [54]. However, gender differences have not typically been examined in recent studies of social avoidance in Western or Chinese Children [16,20,27,28]. According to the Gender Role Stereotype Theory, compared to females, males are traditionally perceived as more dominant during social interaction in China [55,56]. Thus, socially avoidant boys may be less accepted by peers than girls because of this gender stereotype. Therefore, we hypothesized that the relationship between social avoidance and social problems is significant in boys than in girls.

In addition, Chang et al. [43] found that the levels of household chaos are higher among migrant boys than among girls among children and young adolescents in China. Furthermore, for Chinese migrant children, gender moderated the association between household chaos and interpersonal security. Specifically, girls’ interpersonal security is more easily affected by the increasing levels of household chaos than boys [43]. Thus, it seems reasonable to argue that the effect of household chaos may have gender differences in Chinese migrant children. We examine the moderating role of household chaos and gender difference and their interaction effect in the relation between social avoidance and social adjustment in Chinese preschool migrant children.

### 1.4. The Current Study

The primary purpose of the present study was to investigate the moderating role of household chaos between social avoidance and social adjustment among migrant preschool children in China. In addition, we speculated that child gender would also play a moderating role between social avoidance and social adjustment, and the moderating role of household chaos may have gender differences among in the relation between social avoidance and social adjustment. 

Based on past studies, social avoidance was related to maladjustment in Chinese children [16]. This study hypothesized that social avoidance would be positively associated with peer exclusion and internalizing problems, but negatively related to prosocial behavior and interpersonal skills. Furthermore, previous studies have revealed that higher levels of household chaos were associated with children’s negative social adjustment [33]. We hypothesized that household chaos would moderate the relation between social avoidance and social adjustment. Specifically, at higher levels of household chaos, social avoidance was more strongly associated with socioemotional difficulties. 

**Figure 1:**
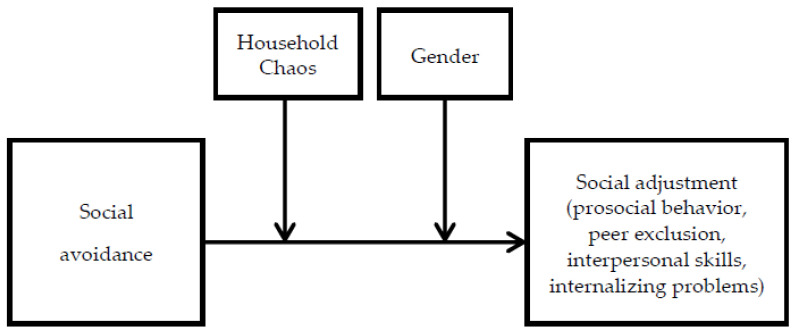
The hypotheses model.

Moreover, we hypothesized that gender would moderate the relationship between social avoidance and social adjustment, and it was anticipated that social avoidance would be more strongly associated with socioemotional maladjustment among boys compared to girls. The relationship is greater in boys than in girls. Moreover, we explored the three-way interactions of social avoidance, household chaos, and gender in the relation between social avoidance and adjustment outcomes among preschool migrant children in China (see Figure 1).

## 2. Methods

### 2.1. Participants 

Participants were 148 migrant preschoolers (82 boys, 66 girls, M_age_ = 62.32 months, SD = 0.50) who were randomly selected from two public kindergartens in Shanghai, People’s Republic of China. Kindergartens in China can be divided into three age groups: primary class (ages 3–4 years), middle class (aged 4–5 years), and advanced class (ages 5–6 years). All participants were of Han ethnicity, representing over 92% of China’s population. Nearly 21.6% of the mothers and 24.3% of the fathers completed high school; 39.9% of the mothers and 27.1% of the fathers completed tertiary education; 35.1% of the mothers and 41.2% of the fathers received bachelor’s degrees, and 3.4% of the mothers and 7.4% of the fathers received postgraduate degrees. 

### 2.2. Measures

#### 2.2.1. Social Avoidance

Children’s mothers completed the Chinese version of Child Social Preference Scale-3 (CSPS-3) [20,32,57]. The current study focused on the subscale for assessing social avoidance, including 4 items (e.g., “My child often goes out of his/her way not to play with other children”; Cronbach’s *α* = 0.74, good construct validity from results of CFA as shown in *χ*^2^ = 0.85, *df* = 2, *χ*^2^/*df* = 0.43, *p* = 0.66, NFI = 1.00, CFI = 1.00, TLI = 1.00, RMSEA = 0.00). Mothers were asked to rate each item on a five-point scale (from 1 = “not at all” to 5 = “a lot”). Higher aggregated scores indicate greater levels of social avoidance. However, considering the similar adjustment patterns among withdrawn Chinese children, it is necessary to control the shared variance of shyness and unsociability when probing the implications of social avoidance [16]. Therefore, mothers were also asked to complete the shyness subscale, which includes 7 items (e.g., “My child seems to want to play with others but is sometimes nervous to”; Cronbach’s *α* = 0.86, good construct validity from results of CFA as shown in *χ*^2^ = 18.96, *df* = 14, *χ*^2^/*df* = 1.35, *p* = 0.17, NFI = 0.99, CFI = 1.00, TLI = 1.00, RMSEA = 0.05), and the unsociability subscale, which comprises 4 items (e.g., “My child often seems content to play alone”; Cronbach’s *α* = 0.64, good construct validity from results of CFA as shown in *χ*^2^ = 11.71, *df* = 2, *χ*^2^/*df* = 5.86, *p* = 0.003, NFI = 0.97, CFI = 0.98, TLI = 0.92, RMSEA = 0.18). All items were rated on a five-point scale (from 1 = “not at all” to 5 = “a lot”). The McDonald’s omega was 0.75, 0.86, and 0.64 for social avoidance, shyness, and unsociality, respectively, and the composite reliability (CR) was 0.89, 0.91, and 0.78, respectively, indicating good reliability. The average variance extracted (AVE) was 0.66, 0.59, and 0.48 for social avoidance, shyness, and unsociability. Thus, the convergent validity was acceptable [58]. The measure has been shown to be reliable and valid in young children in China [32].

#### 2.2.2. Household Chaos

Mothers also completed the Confusion, Hubbub, and Order Scale (CHAOS) [59], which includes 15 items (e.g., “You can’t hear yourself think in our home”, Cronbach’s *α* = 0.75, good construct validity from results of CFA as shown in *χ*^2^ = 88.27, *df* = 90, *χ*^2^/*df* = 0.98, *p* = 0.53, NFI = 0.92, CFI = 1.00, TLI = 1.00, RMSEA = 0.00). Mothers were asked to rate each item with which they agreed with statements describing the chaos in their families on a two-point scale (0 = “true”; 1 = “false”). These items were collected to create the household chaos score, with higher scores indicating greater household chaos. The McDonald’s omega was 0.75. The composite reliability (CR) was 0.90 and the average variance extracted (AVE) was 0.44. For the validity of measurement, Fornell and Larcker’s standards suggest that the average variance extracted (AVE) should exceed 0.5 under ideal conditions, but 0.36~0.5 is acceptable [58]. Thus, the convergent validity was acceptable [58]. The measure has been shown to be reliable and valid in young children in China [60].

#### 2.2.3. Social Adjustment

Teachers were asked to complete the Chinese version of the Child Behavior Scale (CBS) [61,62], including 35 items on a three-point scale (from 1 = “doesn’t apply” to 3 = “certainly applies”). We particularly focused on the subscales assessing prosocial behavior (6 items; e.g., “keeps peers at distance; prefers to play alone”, Cronbach’s *α* = 0.88, good construct validity from results of CFA as shown in *χ*^2^ = 33.05, *df* = 14, *χ*^2^/*df* = 2.36, *p* = 0.003, NFI = 0.99, CFI = 1.00, TLI = 1.00, RMSEA = 0.08), and peer exclusion (7 items; e.g., “ridiculed by peers”, Cronbach’s *α* = 0.93, good construct validity from results of CFA as shown in *χ*^2^ = 13.30, *df* = 14, *χ*^2^/*df* = 0.95, *p* = 0.50, NFI = 1.00, CFI = 1.00, TLI = 1.00, RMSEA = 0.00). The McDonald’s omega was 0.91 and 0.93 for prosocial behavior and peer exclusion, respectively, and composite reliability (CR) was 0.95 and 0.89, respectively, indicating good reliability. The average variance extracted (AVE) was 0.72 and 0.98 for prosocial behavior and peer exclusion, respectively. Thus, the convergent validity was acceptable [58]. The CBS has been proven to be reliable and effective in Chinese young children [62]. 

Teachers also completed the Chinese version of the Social Skills Teacher Rating System (SSTRS) [63,64], which includes 40 items on a three-point scale (from 0 = “never” to 2 = “always”). We focused on the subscales assessing interpersonal skills (11 items; e.g., “Make friends easily”, Cronbach’s *α* = 0.92, good construct validity from results of CFA as shown in *χ*^2^ = 205.00, *df* = 44, *χ*^2^/*df* = 4.66, *p* < 0.001, NFI = 0.98, CFI = 0.98, TLI = 0.98, RMSEA = 0.16) and internalizing problems (4 items; e.g., “Appears lonely”, Cronbach’s *α* = 0.66, good construct validity from results of CFA as shown in *χ*^2^ = 0.19, *df* = 2, *χ*^2^*/df* = 0.10, *p* = 0.91, NFI = 1.00, CFI = 1.00, TLI = 1.02, RMSEA = 0.00). The McDonald’s omega was 0.90 and 0.70 for interpersonal skills and internalizing problems, respectively, and the composite reliability (CR) was 0.67 and 0.56, respectively, indicating good reliability. The average variance extracted (AVE) was 0.96 and 0.83 for interpersonal skills and internalizing problems, respectively [58]. The SSTRS was proven to be reliable and effective in young Chinese children [64]. 

### 2.3. Procedure

The Research Ethics Committee approved the study at Shanghai Normal University. Participants were from two public kindergartens in Shanghai, Eastern China. Participants’ mothers rated children’s social avoidance and household chaos, and teachers were asked to complete the children’s social adjustment measures. The data collection was conducted by a team composed of Chinese preschool education faculty and students. Consent forms were obtained from all participating children and parents through the school. The participation rate was 98%. Data were collected in 2020.

### 2.4. Analytical Strategy

SPSS 23.0 and SPSS macro-PROCESS were used for data analysis [65]. The preliminary investigation involved a series of *t*-tests to explore gender differences and compute the correlations among all main study variables. 

Next, a series of hierarchical regression analyses were conducted to examine social avoidance, household chaos, gender, and two-way and three-way interaction terms as predictors of preschool migrant children’s social adjustment (prosocial behavior, peer exclusion, interpersonal skills, and internalizing problems) in China. In Step 1, child age, shyness, and unsociability were entered as control variables, followed by social avoidance, household chaos, and gender as main effects in Step 2. From Step 3 to Step 5, interaction terms were entered separately to predict each adjustment variable, including Avoidance × Chaos, Avoidance × Gender, and Avoidance × Chaos × Gender. The analysis was conducted for each adjustment variable separately. All continuous predictor variables were standardized [66].

When a significant interaction was found, we then performed a simple slope analysis and plotted the relationships between social avoidance and social adjustment variables in a high value (+1 SD above the mean) and a low value (−1 SD below the mean) of household chaos. The relations between social avoidance and social adjustment in different genders followed the procedure proposed by Aiken and West [66]. 

## 3. Results

### 3.1. Preliminary Analyses

Results from missing data analysis indicated that the missingness for all study variables ranged between 0.7 and 1.4%, and Little’s MCAR test (Little, 1998) suggested that the data were missing completely at random, *χ*^2^(423) = 442.04, *p* > 0.05. The results of the t-tests indicated that there were no gender differences in children’s age (M_boy_ = 62.66, SD = 6.54; M_girl_ = 61.89, SD = 7.05, *t* = 0.68, *p* = 0.50), parental education (M_boy_ = 2.25, SD = 0.78; M_girl_ = 2.27, SD = 0.75, *t* = −0.18, *p* = 0.86), socioeconomic status (M_boy_ = −0.51, SD =2.98; M_girl_ = 0.06, SD = 2.99, *t* = −0.09, *p* = 0.93), shyness (M_boy_ = −1.83, SD = 0.66; M_girl_ = 1.84, SD = 0.72, *t* = −0.09, *p* = 0.93), unsociability (M_boy_ = 1.76, SD = 0.57; M_girl_ = 1.67, SD = 0.58, *t* = 0.93, *p* = 0.35), social avoidance (M_boy_ = 1.31, SD = 0.46; M_girl_ = 1.37, SD = 0.51, *t* = −0.74, *p* = 0.46), internalizing problems (M_boy_ = 0.23, SD = 0.33; M_girl_ = 0.14, SD = 0.25, *t* = 1.71, *p* = 0.10), and household chaos (M_boy_ = 0.24, SD = 0.17; M_girl_ = 0.20, SD = 0.16, *t* = 1.60, *p* = 0.11). Notably, there were significant gender differences in prosocial behavior (M_boy_ = 2.25, SD = 0.57; M_girl_ = 2.45, SD = 0.52, *t* = −2.27, *p* = 0.03), peer exclusion (M_boy_ = 1.23, SD = 0.43; M_girl_ = 1.07, SD = 0.20, *t* = 2.92, *p* = 0.004), and interpersonal skills (M_boy_ = 1.36, SD = 0.44; M_girl_ = 1.57, SD = 0.36, *t* = −3.10, *p* = 0.002).

Table 1 shows the descriptive statistics and correlations of all study variables. As shown in Table 1, social avoidance was significantly and positively correlated with peer exclusion, and significantly and negatively associated with prosocial behavior and interpersonal skills. Household chaos was non-significantly associated with social avoidance and adjustment (i.e., prosocial behavior, peer exclusion, interpersonal skills, and internalizing problems).

### 3.2. Multilevel Models with Interactions

Results of the hierarchical regression models are provided in Table 2. The goal of the following analyses was to explore the potential moderating role of household chaos and gender in the relations between social avoidance and social adjustment. 

#### 3.2.1. Interactions between Social Avoidance and Household Chaos

**Figure 2:**
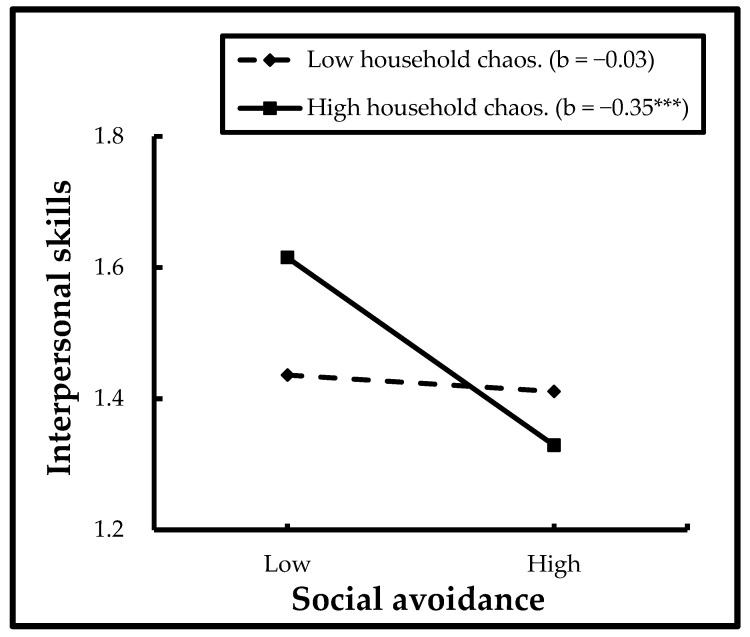
Interactions between social avoidance and household chaos in predicting interpersonal skills. *** *p* < 0.001.

Table 2 indicates that when the effects of age, shyness, and unsociability were controlled, a significant interaction was found in the relation between social avoidance and household chaos in predicting interpersonal skills (β = −0.27, B = −0.22, SE = 0.00, *t* = −2.52, *p* < 0.01). The simple slope effects were examined on social avoidance at high and low values (1 SD above and 1 SD below mean) of household chaos. The results revealed that social avoidance had a negative association with interpersonal skills for children with high levels of household chaos (*b* = −0.30, SE = 0.08, *t* = −4.24, *p* < 0.001); on the contrary, this association was not significant for children with lower levels of household chaos (*b* = −0.03, SE = 0.10, *t* = −0.31, *p* > 0.05) (see Figure 2).

#### 3.2.2. Interactions between Social Avoidance and Gender

**Figure 3:**
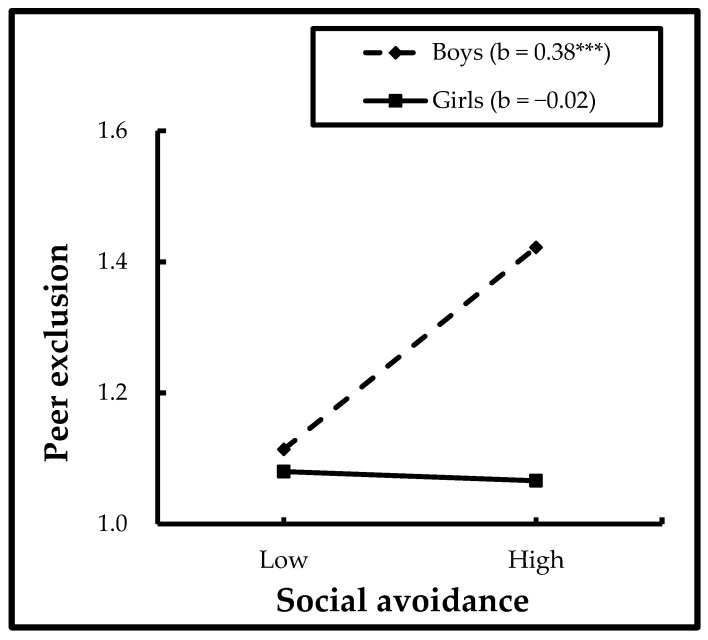
Interactions between social avoidance and gender in predicting peer exclusion. *** *p* < 0.001.

As shown in Table 2, social avoidance significantly interacted with gender in predicting peer exclusion (β = −0.27, B = −0.43, SE = 0.16, *t* = −2.65, *p* < 0.01). Further analyses revealed that social avoidance was significantly related to peer exclusion in boys (*b* = 0.38, SE = 0.08, *t* = 4.82, *p* < 0.001), but not in girls (*b* = −0.02, SE = 0.08, *t* = −0.21, *p* > 0.05) (see Figure 3).

## 4. Discussion

This study extended previous studies by examining the relationship between social avoidance and social adjustment and the moderating role of both household chaos and child gender in a sample of preschool migrant children in China. The result showed that social avoidance is a risk factor for peer exclusion, and household chaos exacerbates the link between social avoidance and interpersonal skills. Moreover, the findings showed that social avoidance significantly predicted boys’ peer exclusion rather than girls. 

### 4.1. Social Avoidance and Social Adjustment

As expected, the findings revealed that after controlling for shyness and unsociability, social avoidance positively predicted peer exclusion among Chinese preschool migrant children. However, Zhu et al. [32] found that among non-migrant preschoolers living in urban areas, social avoidance was not significantly associated with peer exclusion after controlling for shyness and unsociability. These findings seem to suggest that compared with non-migrant socially avoidant preschoolers, migrant socially avoidant preschoolers might face more social adjustment problems. Previous studies found that migrant children likely maintain traditional social behaviors valued in the past of China [30]. Indeed, influenced by Western culture and the large economic and social reforms in China, parents tend to encourage socially avoidant preschoolers to be self-expressional and cooperative to adapt to the urban environment. However, unlike non-migrant families, the social encouragement of migrant parents may increase migrant socially avoidant preschoolers’ risk of peer exclusion, as migrant parents may not have the knowledge and skills to guide their children’s social interactions effectively [67]. Thus, migrant socially avoidant preschoolers without suitable assistance who are encouraged to interact with peers may expose their social problems to peers and cause various social maladjustment including peer exclusion [67]. Moreover, according to Developmental Contextualism [68], during peer interactions, children prefer to select peers with similar behavior and attitude characteristics for communication [69]. Furthermore, the attitudes and behaviors of migrant children are more similar to those of peers in the countryside [70]. Thus, it is relatively challenging for migrant socially avoidant preschool children to receive peer acceptance. These findings expand the previous literature that social avoidance is a risk factor for Chinese children’s social adjustment [16,17].

Our finding indicated that social avoidance was not significantly associated with internalizing problems. After controlling for shyness and unsociability, social avoidance was also not significantly predictive of internalizing problems, which is inconsistent with the previous findings in Western cultures and older Chinese children [20,27,29]. Previous studies revealed that preschoolers tend to be self-centered, and solitary behavior is quite common at this age [13]. Thus, the anxiety and emotional volatility of social avoidance may be overshadowed [32]. Moreover, migrant preschool children constantly face new and unfamiliar territory [5], and there may be many other factors related to internalizing problems. Thus, the unique relationship between social avoidance and internalizing problems may not be exhibited in migrant preschoolers. However, a “cumulative effect” may appear over time, and the unique relationship between social avoidance and internalizing problems may be further exhibited and promoted. Further research is warranted to explore the links between social avoidance and adjustment in Chinese migrant preschoolers.

### 4.2. The Moderating Role of Household Chaos

This study firstly considers the role of household chaos in the relation between social avoidance and social adjustment in preschool migrant children in China. The result showed that household chaos moderates the relationship between social avoidance and interpersonal skills in Chinese migrant preschoolers after controlling for shyness and unsociability, which partially support our moderating hypothesis. Specifically, social avoidance was negatively associated with interpersonal skills among migrant preschoolers with higher levels of household chaos in China. However, the association between social avoidance and interpersonal skills was no longer significant at low levels of household chaos.

Social situations are especially pressured for socially avoidant children; they tend to respond to social situations with great tension [51]. According to Emotional Security Theory, the feeling of security in the family affects children’s adjustment and development [44]. High household chaos was associated with negative parent–child interpersonal relationships, parental negativity, and stressful events [36,45]. Thus, high household chaos produces a toxic family environment, which destroys socially avoidant migrant preschoolers’ emotional security and further prompts social anxiety and fear of interpersonal communication. In addition, according to the Diathesis-Stress Model [46], the sensitivity of socially avoidant migrant preschoolers to negative environments would be intensified. Previous consistent evidence revealed that children who live in an environment with high household chaos might result in more social maladjustments [41,50,55]. Consequently, Chinese preschool migrant children who are exposed to high levels of household chaos may feel more stressed to interact with peers. 

Moreover, compared to socially avoidant urban non-migrant preschoolers, socially avoidant migrant preschoolers may be more likely to be affected by household chaos in China. Previous studies revealed that mothers’ responsivity as a mediator of the association between household chaos and children’s behavior regulation [71] and coordinated and reciprocal mother–child interaction attenuated the association between household chaos and behavior problems in low-income families in young children [39]. In China, migrant parents generally had jobs with long working times that were labor-intensive because of economic pressure [72]. They had less time and energy to accompany children, and the parent–child interactions were characterized as low frequency, time, and initiative [73]. Parents of urban non-migrant children had a higher level of education, and they paid more attention to communication with children [73], which predicted more altruistic behavior, peer competence, and excellent social skills [74,75]. Accordingly, findings from the present study suggest that we should highlight the negative effect of household chaos on social adjustment among socially avoidant migrant preschoolers in China.

### 4.3. The Moderating Role of Gender

The finding revealed that child gender moderates the relationship between social avoidance and social adjustment in preschool migrant children in China. Specifically, social avoidance significantly positively predicts boys’ peer exclusion. In other words, boys with high social avoidance were more likely to have maladjustment, while for girls, social avoidance was not related to peer exclusion.

According to the Biological Theory and Gender Schema Theory [76,77], young children’s behavior has significant gender differences. Boys with a high level of social avoidance may prefer rough, noisy, and large muscle activities when alone [78], and they are more likely to disturb others, resulting in peer exclusion. In contrast, girls enjoy quiet activities [79]. As a result, girls will exhibit more nonverbal behavior if they have a higher level of social avoidance. Although they will not actively interact with peers, this quiet behavior is consistent with children’s gender schema of girls who will not invite peer exclusion. Moreover, according to the Gender Role Stereotype Theory, compared to females, males are traditionally perceived as more dominant during social interaction in China [54,55]. Thus, socially avoidant boys may be less accepted by peers than girls.

### 4.4. Limitations and Future Directions

Some shortcomings remained in the current study. First, as the preschoolers were too young to complete the written questionnaires, all the data collection relied on the mother-report and teacher-report, which may result in social desirability bias. Although this method collected data from the typical resources (i.e., at home and in the classroom) for preschoolers, and our measurements demonstrated validity, the data may still be affected by the subjective factors of test subjects. Future studies could use more self-report methods, including child interviews and peer evaluation with age-appropriate rating items. Secondly, our study clarified the relationships among household chaos, social avoidance, and social adjustment. As our study adopted a cross-sectional design, the causality and the directions of relations among variables cannot be supported and inferenced. For example, when experiencing peer exclusions, the children’s level of social avoidance may be further promoted over time [19]. In future studies, we could adopt a longitudinal design to disentangle the temporal order of these variables. Thirdly, our study was conducted in one of China’s largest and most developed cities. Considering the unequal paces of China’s social and economic development, we should be cautious when extending the results to other regions such as small cities. Therefore, we can conduct research in other cities with different economic and cultural backgrounds in the future. Finally, the sample size of this study was small because of the limited time. Future studies could increase the time of data collection and expand the sample size.

Despite these limitations, the current study’s findings greatly contribute practical significance to socially avoidant migrant children in China. On the one hand, household chaos plays a significant role in developing social competence. Specifically, higher levels of household chaos mean negative interpersonal skills. Therefore, parents should pay more attention to the family environment, such as noise, daily routine, and crowded situations, for example, making less noise from TVs or mobile phones by using headphones; tidying up clutter regularly to ensure orderliness. On the other hand, more attention should be paid to socially avoidant boys, compared to girls, who are more likely to have peer exclusion. Therefore, the teacher could pay particular attention to whether boys exhibit aggressive behavior when encouraging socially withdrawn children to interact with peers.

## 5. Conclusions

In summary, the current study revealed that social avoidance significantly predicted peer exclusion between young Chinese migrant children. Additionally, household chaos moderated the relationship between social avoidance and social adjustment. Specifically, social avoidance was negatively associated with interpersonal skills at high levels of household chaos. In contrast, social avoidance was not associated with interpersonal skills at a low level of household chaos. As well, there are gender differences in the relationships between social avoidance and social adjustment. Specifically, compared with girls, social avoidance was positively associated with peer exclusion among boys. 

## Figures and Tables

**Table 1:** Descriptive statistics and inter-correlations for all study variables (*n* = 148).

	1	2	3	4	5	6	7	8	9	10	11	12
1. Gender	-											
2. Child age	−0.06	-										
3. Parental education	0.02	0.04	-									
4. Socioeconomic status	0.02	0.01	0.90 ***	-								
5. Shyness	0.01	−0.07	0.02	0.04	-							
6. Unsociability	−0.08	−0.10	0.07	0.08	0.63 ***	-						
7. Social avoidance	0.06	−0.10	0.07	0.07	0.57 ***	0.62 ***	-					
8. Prosocial behavior	0.19 *	0.46 ***	0.10	0.07	−0.27 **	−0.29 ***	−0.20 **	-				
9. Peer exclusion	−0.22 **	0.01	0.04	0.01	0.20 *	0.25 **	0.23 **	−0.51 ***	-			
10. Interpersonal skills	0.25 **	0.19 *	0.08	0.10	−0.30 ***	−0.32 ***	−0.26 **	0.60 ***	−0.65 ***	-		
11. Internalizing problems	−0.14	0.10	0.09	0.02	0.24 **	0.15	0.14	−0.25 **	0.35 ***	−0.42 ***	-	
12. Household chaos	−0.13	0.07	0.06	0.00	0.08	0.07	0.16	0.05	0.13	0.01	0.10	-
M	-	62.32	-	-	1.84	1.72	1.33	2.34	1.16	1.45	0.19	0.22
SD	-	6.76	-	-	0.68	0.57	0.48	0.56	0.36	0.42	0.30	0.17

Note. * *p* < 0.05, ** *p* < 0.01, *** *p* < 0.001.

**Table 2:** Effects of social avoidance, household chaos, and gender in relation to indices of social adjustment.

	B	SE	*t Value*	*95%CI*	*R* ^2^	△*R*^2^	△*F*
**Prosocial behavior**							
Child age	0.07	0.01	6.32 ***	[−0.04, 0.09]			
Shyness	−0.14	0.10	−1.55	[−0.33, 0.04]			
Unsociability	−0.16	0.10	−1.58	[−0.35, 0.04]			
Social avoidance	0.00	0.13	0.01	[−0.26, 0.26]			
Household chaos	0.08	0.07	1.14	[−0.06, 0.22]			
Gender	0.38	0.14	2.68 *	[0.10, 0.67]			
Avoidance × Chaos	−0.14	0.08	−1.78	[−0.30, 0.02]	0.34	0.01	1.98
Avoidance × Gender	0.08	0.15	0.51	[−0.22, 0.37]	0.34	0.00	0.59
Avoidance × Chaos × Gender	0.20	0.13	1.49	[−0.06, −0.02]	0.36	0.01	2.23
**Peer exclusion**							
Child age	0.00	0.01	−0.31	[−0.20, −0.03]			
Shyness	0.03	0.10	0.32	[−0.17, 0.24]			
Unsociability	0.11	0.11	1.04	[−0.10, 0.33]			
Social avoidance	0.32	0.14	2.20 *	[0.03, 0.60]			
Household chaos	0.05	0.08	0.65	[−0.11, 0.21]			
Gender	−0.38	0.16	−2.43 *	[−0.70, −0.07]			
Avoidance × Chaos	0.15	0.09	1.64	[−0.03, 0.32]	0.15	0.02	4.00
Avoidance × Gender	−0.43	0.16	−2.65 **	[−0.75, −0.11]	0.20	0.05	8.49
Avoidance × Chaos × Gender	−0.20	0.15	−1.38	[−0.50, 0.09]	0.21	0.01	1.89
**Interpersonal skills**							
Child age	0.02	0.01	2.15 *	[0.00, 0.05]			
Shyness	−0.14	0.10	−1.45	[−0.34, 0.05]			
Unsociability	−0.16	0.11	−1.49	[−0.37, 0.05]			
Social avoidance	−0.05	0.14	−0.38	[−0.33, 0.22]			
Household chaos	0.10	0.08	1.33	[−0.05, 0.25]			
Gender	0.50	0.15	3.27 **	[0.20, 0.80]			
Avoidance × Chaos	−0.22	0.09	−2.64 **	[−0.39, 0.06]	0.26	0.04	8.50
Avoidance × Gender	0.05	0.16	0.30	[−0.26, 0.36]	0.26	0.00	0.21
Avoidance × Chaos × Gender	0.13	0.14	0.89	[−0.15, 0.41]	0.26	0.00	0.80
**Internalizing problems**							
Child age	0.02	0.01	1.26	[−0.01, 0.04]			
Shyness	0.24	0.11	2.24 *	[0.03, 0.46]			
Unsociability	0.03	0.12	−0.27	[−0.26, 0.20]			
Social avoidance	0.21	0.15	1.38	[−0.09, 0.51]			
Household chaos	0.05	0.08	0.60	[−0.12, 0.22]			
Gender	−0.28	0.17	−1.70 †	[−0.62, 0.05]			
Avoidance × Chaos	−0.08	0.09	−0.82	[−0.26, 0.11]	0.10	0.00	0.07
Avoidance × Gender	−0.32	0.17	−1.83	[−0.66, 0.03]	0.11	0.02	2.86
Avoidance × Chaos × Gender	0.16	0.15	1.00	[−0.15, 0.46]	0.12	0.01	1.01

Note. * *p* < 0.05; ** *p* < 0.01; *** *p* < 0.001; † *p* < 0.10.

## Data Availability

The data presented in this study are available on request from the corresponding author.
